# Celastrol Ameliorates Ulcerative Colitis-Related Colorectal Cancer in Mice via Suppressing Inflammatory Responses and Epithelial-Mesenchymal Transition

**DOI:** 10.3389/fphar.2015.00320

**Published:** 2016-01-13

**Authors:** Lianjie Lin, Yan Sun, Dongxu Wang, Shihang Zheng, Jing Zhang, Changqing Zheng

**Affiliations:** Department of Gastroenterology and Hepatology, Shengjing Hospital of China Medical UniversityShenyang, China

**Keywords:** celastrol, ulcerative colitis, colorectal cancer, inflammation, epithelial-mesenchymal transition, oncologic proteins

## Abstract

Celastrol, also named as tripterine, is a pharmacologically active ingredient extracted from the root of traditional Chinese herb *Tripterygium wilfordii* Hook F with potent anti-inflammatory and anti-tumor activities. In the present study, we investigated the effects of celastrol on ulcerative colitis-related colorectal cancer (UC-CRC) as well as CRC *in vivo* and *in vitro* and explored its underlying mechanisms. UC-CRC model was induced in C57BL/6 mice by administration of azoxymethane (AOM) and dextran sodium sulfate (DSS). Colonic tumor xenograft models were developed in BALB/c-nu mice by subcutaneous injection with HCT116 and HT-29 cells. Intragastric administration of celastrol (2 mg/kg/d) for 14 weeks significantly increased the survival ratio and reduced the multiplicity of colonic neoplasms compared with AOM/DSS model mice. Mechanically, celastrol treatment significantly prevented AOM/DSS-induced up-regulation of expression levels of oncologic markers including mutated p53 and phospho-p53, β-catenin and proliferating cell nuclear antigen (PCNA). In addition, treatment with celastrol inhibited inflammatory responses, as indicated by the decrease of serum tumor necrosis factor-α (TNF-α), interleukin (IL)-1β and IL-6, down-regulation of cyclooxygenase-2 (COX-2) and inducible nitric oxide synthase (iNOS), and inactivation of nuclear factor κB (NF-κB). Moreover, celastrol obviously suppressed epithelial-mesenchymal transition (EMT) through up-regulating E-cadherin and down-regulating N-cadherin, Vimentin and Snail. Additionally, we also demonstrated that celastrol inhibited human CRC cell proliferation and attenuated colonic xenograft tumor growth via reversing EMT. Taken together, celastrol could effectively ameliorate UC-CRC by suppressing inflammatory responses and EMT, suggesting a potential drug candidate for UC-CRC therapy.

## Introduction

Ulcerative colitis (UC) is a chronic and non-specific inflammatory bowel disease characterized by ulcer and erosion of the rectum and colon (Khor et al., 2011). Epidemiological studies have shown that UC is one of the three highest risk factors for developing colorectal cancer (CRC) due to delayed healing and chronic inflammation (Eaden et al., 2001). CRC is the second most common cancer in women and the third in men worldwide (Ferlay et al., 2010). Although UC-associated CRC (UC-CRC) accounts for only 1–2% of all CRC cases in the general population, it is considered as one of the most serious complications of UC and accounts for approximately 10–15% of all deaths in UC patients (Lakatos and Lakatos, 2008). However, so far, there is no specific and effective treatment for UC and UC-CRC. Therefore, to explore novel drugs with high efficacy and low toxicity against UC and UC-CRC is very imperative and significant.

Celastrol, a triterpene, is a pharmacologically active ingredient extracted from the traditional Chinese medicinal plant *Tripterygium wilfordii* Hook F (also named as Thunder of God Vine) and exhibits significant activities in the treatment of chronic inflammatory, autoimmune diseases, cancer, and neurodegenerative diseases (Allison et al., 2001; Dai et al., 2010; Ge et al., 2010; Venkatesha et al., 2011; Wong et al., 2012). Recently, Shaker et al. (2014) have reported that celastrol ameliorates dextran sulfate sodium (DSS)-induced colitis in mice via modulating intestinal epithelial homeostasis, colonic oxidative stress, and inflammatory cytokines. Meanwhile, several studies have demonstrated that celastrol induces apoptosis in human CRC cells through up-regulation of death receptors and β-catenin pathway and suppresses invasion through down-regulation of CXCR4 chemokine receptor (Sung et al., 2010; Yadav et al., 2010; Lu et al., 2012). The above evidence led us to investigate whether or not celastrol could prevent UC-CRC and if so, through what mechanism.

Chronic inflammation plays a crucial role in the procession of UC tumorigenesis through the induction of cellular DNA damage, telomere shortening, and senescence (Risques et al., 2011). Various initiating factors have been found to be involved in cancer-related inflammation such as nuclear factor κB (NF-κB), tumor necrosis factor-α (TNF-α), interleukin (IL)-1β, and IL-6 (Zhu et al., 2013). The epithelial-mesenchymal transition (EMT) is a process characterized by the loss of epithelial cell markers including E-cadherin, and the acquisition of a mesenchymal phenotype with expression of mesenchymal proteins such as Vimentin, which serves important functions in tumor initiation, progression, invasion, and metastasis (Guarino et al., 2007; Thiery et al., 2009; Chen et al., 2013). Recent studies have demonstrated that EMT also contributes to the pathogenesis of UC and colorectal carcinogenesis, and those factors involved in the development of inflammation are also crucial for the signaling pathways of EMT (Zhu et al., 2013; Tahara et al., 2014). Thus, we hypothesized that the natural agent celastrol might be a promising candidate for the treatment of UC-CRC via suppressing inflammatory responses and epithelial-mesenchymal transition.

In the present work, to test this hypothesis, we developed an azoxymethane (AOM)/DSS-induced UC-CRC mouse model, and demonstrated that celastrol effectively alleviated UC-CRC via suppressing inflammatory response and EMT. In parallel, the *in vivo* and *in vitro* anti-tumor activities and molecular mechanisms of celastrol in human CRC cell lines and xenograft mouse models were further determined. Here, our findings suggest that celastrol has potentials in the treatment of UC-CRC and provide new useful clues regarding its possible mechanisms.

## Materials and Methods

### Cells, Animals, and Materials

Human colorectal adenocarcinoma cell lines HCT116 and HT-29 were obtained from Shanghai Institute of Cell Resource Center of Life Science (Shanghai, China). All cells were cultured in McCOY’s 5A medium (Sigma-Aldrich, St. Louis, MO, USA) supplemented with 10% fetal bovine serum (FBS; Hyclone, USA), 100 mg/ml streptomycin and 100 U/ml penicillin at 37°C in humidified atmosphere with 5% CO_2_.

Male C57BL/6 mice (*n* = 65, 6–8 weeks old) used for UC-CRC models and male BALB/c-nu mice (*n* = 36, 5 weeks old) used for colorectal tumor xenograft models were purchased from Vital River Laboratory Animal Technology Co. Ltd (Beijing, China). All animals were housed under controlled conditions (temperature 22 ± 1°C, humidity 40–60% and 12 h dark/light cycle) and free access to a standard laboratory diet and water for 2 weeks. All animal care and experimental procedures were carried out in accordance with the recommendation of the Animal Care Ethics and Use Committee of China Medical University and approved by this Committee.

Celastrol (≥98%) was purchased from Dalian Melone pharmaceutical Co., Ltd (Dalian, China). For *in vitro* studies, celastrol was dissolved in dimethyl sulfoxide (DMSO; Sigma–Aldrich) at a stock concentration of 44 mM. For animal experiments, celastrol was dissolved in DMSO at 20 mg/ml and then diluted with 0.9% saline to the final concentrations (1% DMSO) before administration.

### Development of UC-CRC Model and Treatment Procedure

**FIGURE 1:**
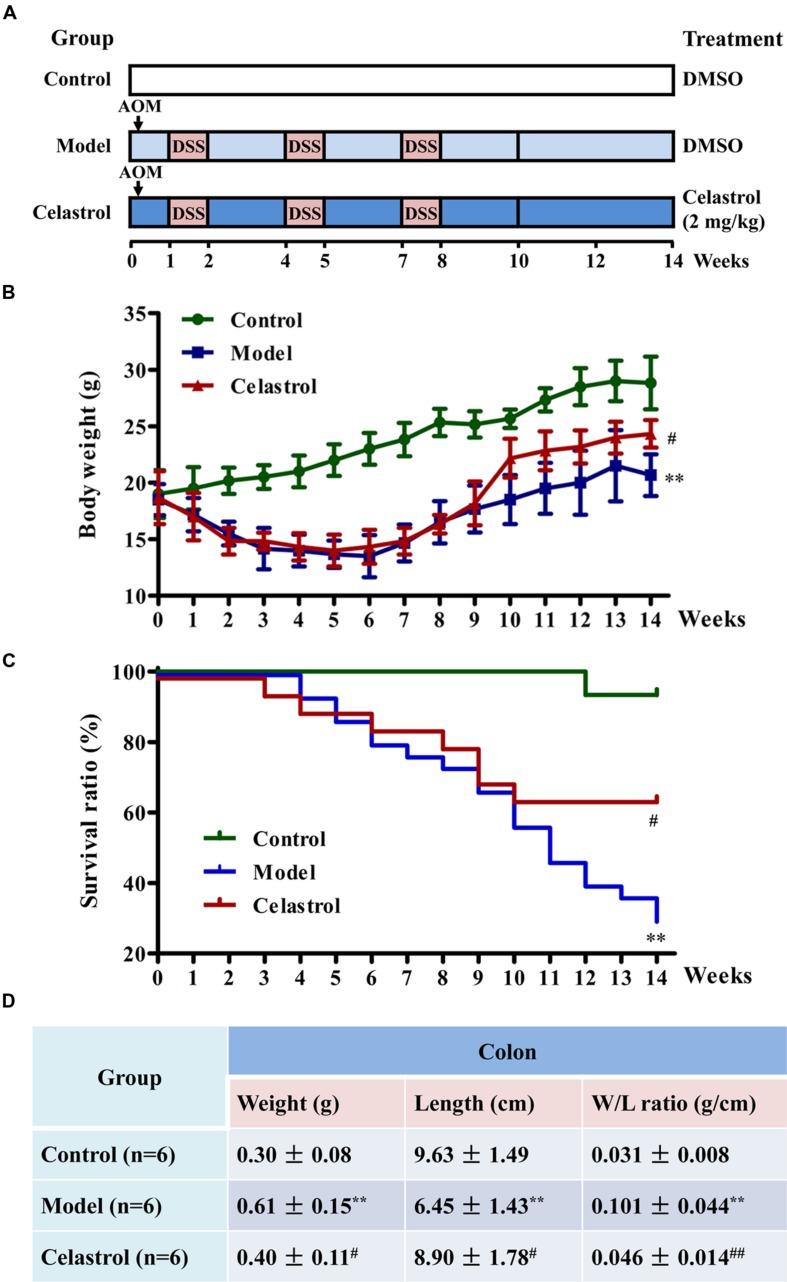
**Effects of celastrol on the general health and survival of mice treated with azoxymethane (AOM) and dextran sodium sulfate (DSS).** **(A)** Experimental protocol for ulcerative colitis-related colorectal cancer (UC-CRC) model and treatment. The details were described in the section “Materials and Methods.” **(B)** Effect of celastrol (2 mg/kg) on body weight of mice. Body weight of each mouse was measured once per week. **(C)** Effect of celastrol (2 mg/kg) on survival ratio of mice. Survival status of each mouse was recorded every week. Fifteen mice in the control group, 30 mice in model group, and 20 mice in celastrol group. **(D)** Effect of celastrol (2 mg/kg) on colon weight and colon length. At the end of experiment, colon tissues were removed and the weight and length were measured. Data are presented as mean ± SD. ^∗∗^*p* < 0.01 vs. the control group; ^#^*p* < 0.05 and ^##^*p* < 0.01 vs. the AOM/DSS model group.

The procedures of induction of UC-CRC model by AOM and DSS were presented in **Figure 1A**. In the study, 65 mice were randomly divided into three groups: 15 mice in the control group, 30 mice in model group (AOM/DSS), and 20 mice in celastrol group (AOM/DSS + celastrol treatment). To develop the UC-CRC model, the mice were given a single intraperitoneal injection of AOM (10 mg/kg body weight in 0.9% saline, Sigma–Aldrich) at first week following adaptation. One week later, the animals were given 3% DSS (Mpbio, Solon, OH, USA) added to the drinking water for 7 days followed by 14 days of drinking water for recovery, and this cycle was repeated twice. Celastrol (2 mg/kg/d) or the vehicle (1%, v/v, DMSO in normal saline) was administrated by gavage daily from first week until the end of 14th week. During the total experimental periods, body weights and survival ratio were measured every week. At the end of the experiment, blood was collected for ELISA, then mice were sacrificed and colon tissues were removed. After measuring the weight and length, the colons were slit open longitudinally along the main axis and washed with phosphate buffer saline (PBS, pH 7.4). The number of tumors in the colons was recorded, and the diameter of each tumor was measured using a sliding caliper, then total tumor area of each colon was calculated. Subsequently, some colon tissues were fixed in 4% paraformaldehyde buffer for further histopathological examination and immunohistochemical analysis, while others were flash-frozen in liquid nitrogen and kept at -80°C for western blotting analysis.

### Histopathological Examination

For histopathology analysis, paraformaldehyde fixed colonic tissues were dehydrated in gradient alcohol, embedded in paraffin and cut into serial sections at 5 μm. Then, these sections were stained with haematoxylin and eosin (H&E) solution and observed under an optical microscope (DP73, OLUMPUS, Japan). Pathological assessment was performed independently and blindly by two pathologists.

### Immunohistochemical Staining

For immunohistochemical examination, paraffin-embedded colonic sections were deparaffinized in xylene and hydrated in gradient alcohol. Then, antigen retrieval was performed by heating in pre-boiling buffer in a microwave for 10 min. Next, slides were incubated in 3% hydrogen peroxide solution for 15 min to quench endogenous peroxidase activity and then blocked by 10% goat serum in PBS (pH 7.4) for 15 min at room temperature. Subsequently, slides were incubated with primary antibodies in a humidified chamber at 4°C overnight: cyclooxygenase-2 (COX-2; 1:300), inducible nitric oxide synthase (iNOS; 1:300), β-catenin (1:200), E-cadherin (1:200), N-cadherin (1:200; BOSTER, Wuhan, China), proliferating cell nuclear antigen (PCNA; 1:100), Vimentin (1:300), Snail (1:100; Bioss, Beijing, China), or p53 (1:50), p-p53 (1:50; Santa Cruz, Dallas, TX, USA). After incubation with biotinylated goat anti-rabbit secondary antibody (1:200; Beyotime, Jiangsu, China) and avidin-biotin-horseradish peroxidase (HRP; Beyotime, Jiangsu, China), slides were visualized using diaminobenzidine (DAB), counterstained with haematoxylin and observed under an optical microscope.

### Enzyme-Linked Immunosorbent Assays (ELISA) for TNF-α, IL-1β, and IL-6

The levels of TNF-α, IL-1β, and IL-6 in the serum were measured using commercial Mouse TNF-α, IL-1β, and IL-6 ELISA Kits (BOSTER, Wuhan, China), respectively, according to the manufacturer’s protocols. Briefly, 100 μl diluent standard or sample serum was added into the antibody-coated wells and incubated for 90 min at 37°C. After washing, samples were incubated with the biotinylated polyclonal antibody for 60 min at 37°C. Then, 100 μl avidin-peroxidase complex solution was added and incubated for 30 min at 37°C. After washing, 90 μl 3,3′,5,5′-Tetramethylbenzidine (TMB) color liquid was added, and the mixture was protected from light for 30 min at 37°C. Finally, 100 μl stop solution was pipetted to stop the reaction, and the optical density was determined at 450 nm using a plate reader (ELX-800, BIOTEK, USA).

### Cell Viability Assays

MTT assay was used to measure the anti-proliferative effect of celastrol on two kinds of CRC cell lines HCT116 and HT-29. Cells were seeded in 96-well plates at a density of 3000 cells/well and were allowed to attach for overnight. Then cells were treated with 0–40 μM celastrol for 24 and 48 h. After the treatment, 20 μl of MTT (5 mg/ml, Sigma–Aldrich) dissolved in PBS was added to each well and incubated at 37°C for 4 h. Subsequently, the media with MTT were removed and the formazan granules generated by live cells were dissolved in 200 μl DMSO. The absorbance at 490 nm was measured using a plate reader.

### Human Colorectal Tumor Xenograft Model and Treatment

HT-29 or HCT-116 cells (1 × 10^7^) suspended in 0.2 ml of serum-free McCOY’s 5A medium were inoculated subcutaneously into the right flank of male 5-week-old BALB/c nude mice. The tumor diameters were measured with digital caliper every 3 days and their volumes were calculated following a standard formula: length × width^2^/2. On 10th day after inoculation, for two colorectal tumor models, mice were, respectively, randomized into three groups (*n* = 6) and treated with either vehicle (model group) or celastrol (1 mg/kg or 2 mg/kg) by gavage daily for the duration of the experiment (18 days). By the end of the experiment, mice were sacrificed, and then all tumor xenografts were removed and measured followed by being flash-frozen in liquid nitrogen and kept at -80°C for western blotting analysis.

### Cytoplasmic and Nuclear Protein Extraction

Nuclear proteins and cytoplasmic proteins were extracted from the colon tissues using the Nuclear and Cytoplasmic Protein Extraction Kit (Beyotime, Jiangsu, China) according to the manufacturer’s instructions. Briefly, colon tissues were cut into small pieces and homogenized with cytoplasmic protein extraction agent A and B. After centrifuging at 1500* g* for 5 min at 4°C, the supernatant was collected as partial cytoplasmic protein and the pellet was dissolved with cytoplasmic protein extraction agent A supplemented with PMSF. After incubation on ice for 15 min, cytoplasmic protein extraction agent B was added and incubated for 1 min on ice. Then, the samples were centrifuged at 12,000 *g* for 5 min at 4°C, and the supernatant was combined with the above cytoplasmic protein. The pellet was re-suspended in nuclear extraction buffer supplemented with PMSF on ice for 30 min and the supernatant containing the nuclear protein were obtained following centrifuging at 12,000 *g* for 10 min at 4°C. All protein extracts were stored at -80°C.

### Western Blot Analysis

Colon tissues and tumor tissues were lysed in RIPA buffer (Beyotime, Jiangsu, China) with protease and phosphatase inhibitors on ice for 1 h. The lysates were centrifuged at 12,000 *g* for 10 min at 4°C and the supernatant was collected as the total lysate protein. HCT116 and HT-29 cells were treated with celastrol (0–40 μM) for 48 h, then were harvested and lysed in RIPA buffer supplemented with PMSF and phosphatase inhibitors on ice for 1 h. After centrifuging the cell suspension at 12,000 *g* for 10 min at 4°C, the suspension was collected as the whole cell protein. The protein concentration was determined with BCA Protein Assay Kit (Beyotime, Jiangsu, China) and a plate reader according to the manufacturer’s instructions.

For Western blot analysis, 40 μg of protein from each sample was separated by electrophoresis on 8–13% PAGE-1% SDS gels, and transferred onto polyvinylidene difluoride membranes (Millipore, Billerica, MA, USA). After blocking with 5% non-fat milk in TBST (0.1%) for 1 h at room temperature, the membranes were incubated with appropriate primary antibody overnight at 4°C: COX-2 (1:400), iNOS (1:400), NF-κB p65 (1:500), β-catenin (1:400), E-cadherin (1:400), N-cadherin (1:400; BOSTER, Wuhan, China), PCNA (1:500), Vimentin (1:500), Snail (1:500; Bioss, Beijing, China), or p53 (1:200), p-p53 (1:200; Santa Cruz, Dallas, TX, USA). Then, the blots were washed four times for 5 min each in TBST and incubated with secondary HRP-conjugated goat anti-rabbit or anti-mouse IgGs (1:5000; Beyotime, Jiangsu, China) for 45 min at 37°C. The interest proteins were visualized using enhanced chemiluminescence (ECL; 7Sea, Shanghai, China) and the densitometry of band was analyzed through Gel-Pro-Analyzer system (Liuyi, Beijing, China). Equal loading of protein was confirmed by stripping the blots and re-probing with Histone H3 (1:500; Bioss, Beijing, China) or β-actin antibody (1:1000; Santa Cruz, Dallas, TX, USA). Nuclear NF-κB p65 band densities were normalized to Histone H3, while other band densities were normalized to β-actin.

### Statistical Analysis

Data were presented as mean ± SD (standard deviation) of three independent experiments unless otherwise specified. All statistical analyses were performed using GraphPad Prism Software Version 5.0 (GraphPad Software Inc., La Jolla, CA, USA). Data between two groups were compared with two-tailed independent *t*-test and data from more than three groups were analyzed by One-Way ANOVA followed by Bonferroni test. Counting data were analyzed with non-parametric test (Mann–Whitney test). Kaplan–Meier survival analysis was used to evaluate the survival ratio. *p* < 0.05 was considered as statistical significance.

## Results

### Celastrol Improves the General Health and Survival of Mice Treated with AOM/DSS

As shown in **Figure 1B**, body weight loss was significant in mice treated with AOM in combination with three cycles of DSS during the experimental period compared with the control mice. However, this symptom was alleviated in mice treated with celastrol (2 mg/kg) during the recovery periods when they received tap water without DSS. According to the Kaplan–Meier survival curves (**Figure 1C**), celastrol treatment also significantly increased the survival ratio of AOM/DSS-treated mice from 11th week to the end of experiment. In agreement with previous studies (Li et al., 2010), exposure to AOM and DSS caused a significant increase in colon weight and decrease in colon length, which was considered as a result of apparent mucosal thickening. Notably, such remarkable increase in colon weight to colon length ratio in mice receiving AOM and DSS was significantly reduced by celastrol treatment (**Figure 1D**).

### Celastrol Reduces the Multiplicity of Colonic Neoplasms and the Expression of Oncogenic Proteins

**FIGURE 2:**
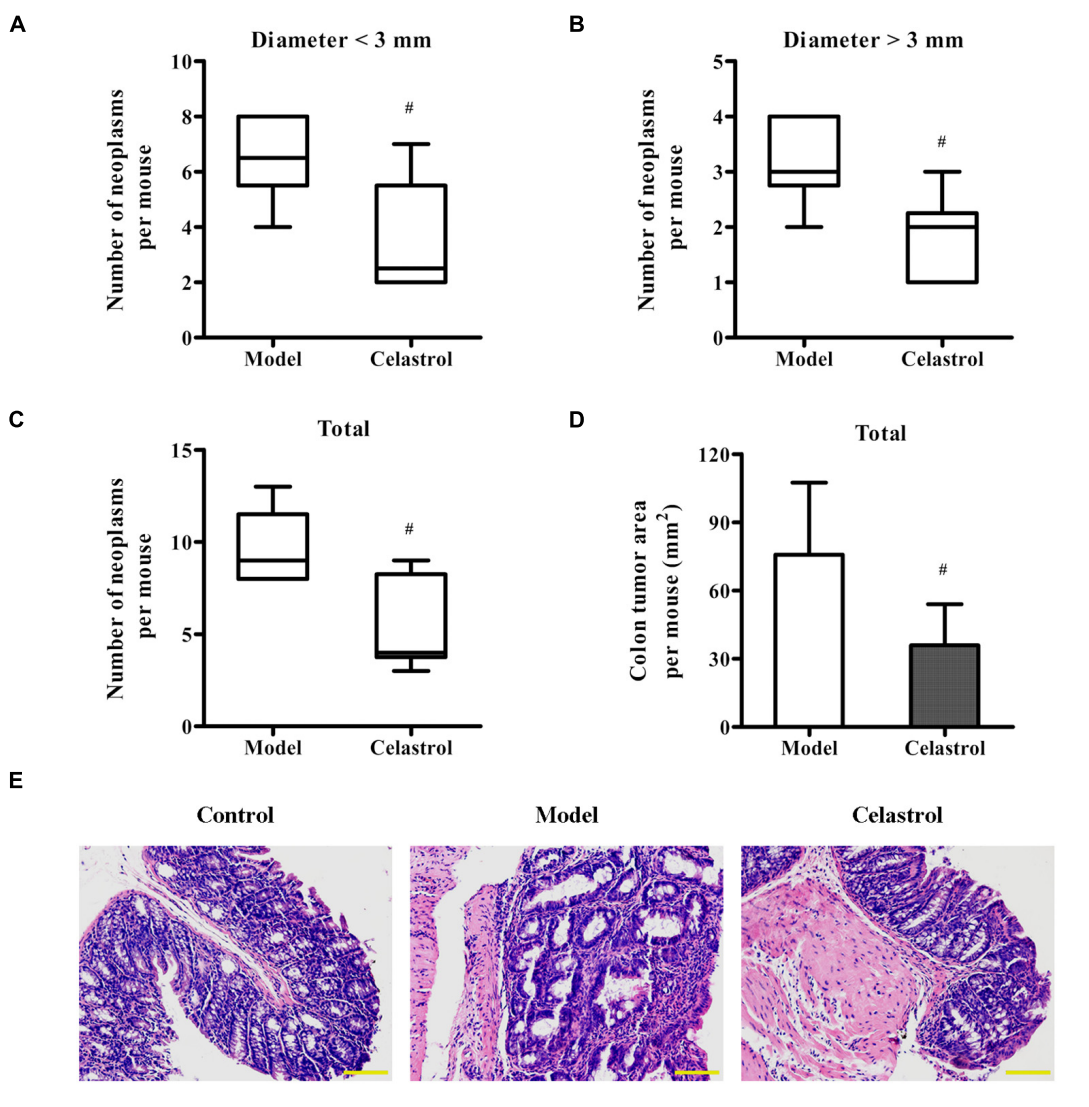
**Effects of celastrol on the burden of colonic neoplasms in AOM/DSS-treated mice.** Colon tissues were removed, the number and size of tumors in each colon was measured, and the tumor area was calculated. **(A,B)** Effect of celastrol (2 mg/kg) on multiplicity of colonic neoplasms in different sizes (diameter > 3 mm and diameter < 3 mm). **(C)** Effect of celastrol (2 mg/kg) on the total number of tumors per mouse. **(D)** Effect of celastrol (2 mg/kg) on the total tumor area per mouse. **(E)** Representative colonic sections from the control mice, AOM/DSS model mice, and AOM/DSS in combination with celastrol (2 mg/kg) treated mice were stained with haematoxylin and eosin (H&E) for histological assessment. Original magnification was 200×. Data are presented as mean ± SD (*n* = 6). ^#^*p* < 0.05 vs. the AOM/DSS model group.

Treatment with AOM and DSS led to 100% incidence of colonic neoplasms with multiplicity of 9.67 ± 2.07 per mouse in model group. Although celastrol administration (2 mg/kg) failed to reduce the incidence of colonic neoplasms, not only did celastrol treatment significantly decrease the number of small neoplasms (diameter < 3 mm) but also the number of large neoplasms (diameter > 3 mm; **Figures 2A,B**). Additionally, celastrol led to an over 40% reduction in the number of total tumors and a more than 50% decrease in tumor area (**Figures 2C,D**). Histologically, crypt destruction, inflammatory cell infiltration, and colon epithelial hyperplasia were observed in the tumor-adjacent colon tissues of AOM/DSS-treated mice. Nevertheless, these symptoms were remarkably mitigated in mice receiving celastrol (**Figure 2E**). There was no colonic tumor observed in the control group.

**FIGURE 3:**
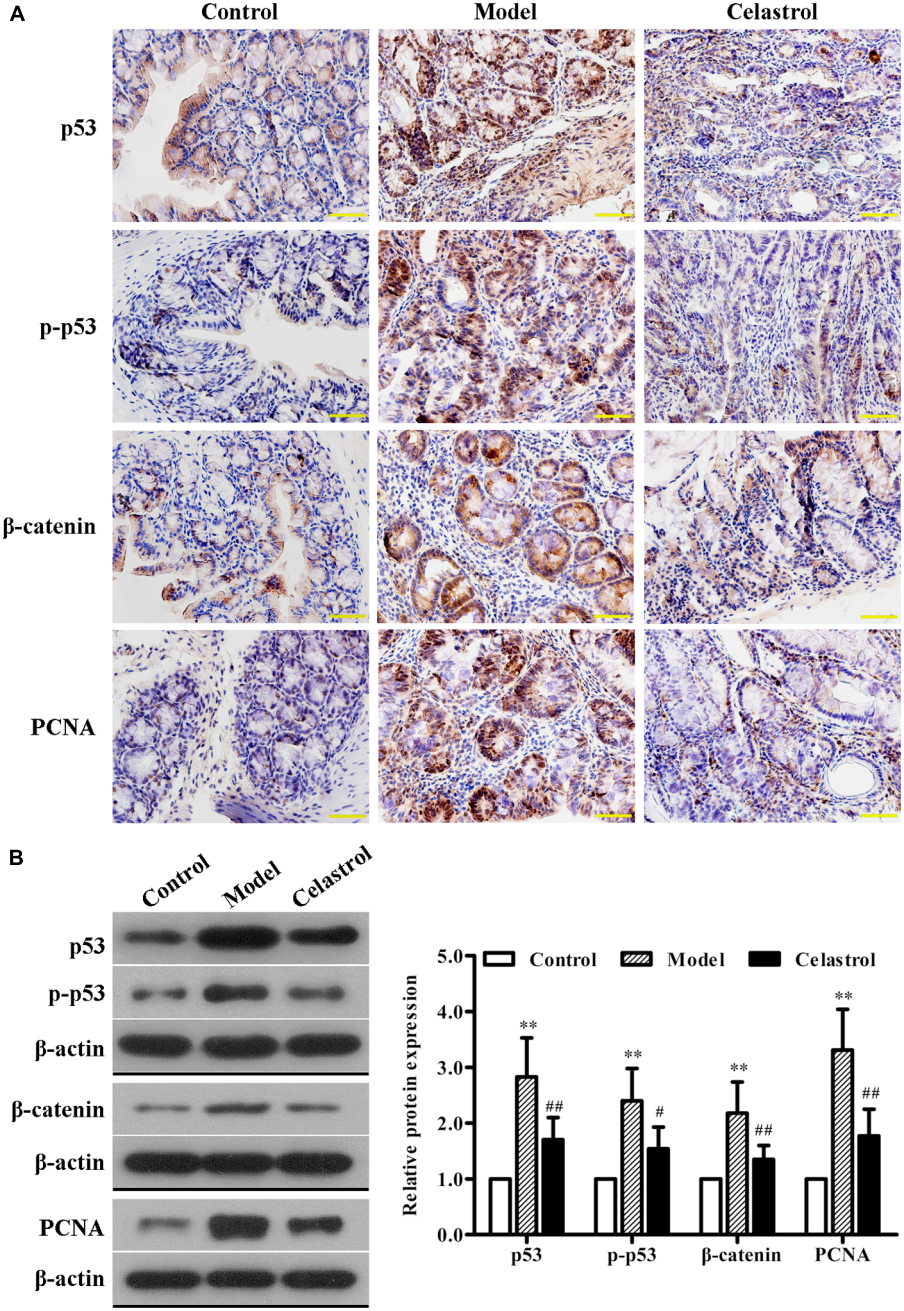
**Effects of celastrol (2 mg/kg) on the oncogenic protein expression in AOM/DSS-treated mice.** **(A)** The expression of p53, p-p53, β-catenin, and proliferating cell nuclear antigen (PCNA) in normal colonic tissues or colonic tumor tissues was evaluated using immunohistochemical staining. Shown are representative section of colon tissues from the control group, UC-CRC model group and celastrol-treated group. Original magnification was 400×. **(B)** The expression levels of p53, p-p53, β-catenin, and PCNA in colonic tissues from each group were determined by western blot analysis. Representative bands are shown (left), and the relative band intensity ratio was analyzed (right). Data are presented as mean ± SD (*n* = 6). ^∗∗^*p* < 0.01 vs. the control group; ^#^*p* < 0.05 and ^##^*p* < 0.01 vs. the AOM/DSS model group.

We also determined the expression levels of neoplastic markers by immunohistochemistry and western blotting. Existing evidence indicate that p53 mutation is an early event of UC-CRC progression, which has been shown to be present in approximately 50% of patients with UC-CRC (Lashner et al., 2003). In general cases, the immunohistochemical staining and western blot of p53 mainly represent the accumulated mutated proteins due to the much longer half-life of mutated p53 than the active wild-type protein (Lashner et al., 2003). As shown in **Figures 3A,B**, the expression levels of p53 and p-p53 proteins in colonic neoplasms of AOM/DSS model mice were significantly increased compared with the control group, implicating the involvement of p53 mutation in our UC-CRC model. Additionally, the expression of oncologic proteins β-catenin and PCNA were dramatically up-regulated in model group. These changes suggested that AOM/DSS-induced UC-CRC was phenotypically similar to human UC-CRC. More noteworthy was that such increase in the expression levels of these neoplastic markers induced by AOM/DSS was significantly suppressed by celastrol treatment (2 mg/kg).

### Celastrol Inhibits Inflammatory Responses in AOM/DSS-Induced Mice

**FIGURE 4:**
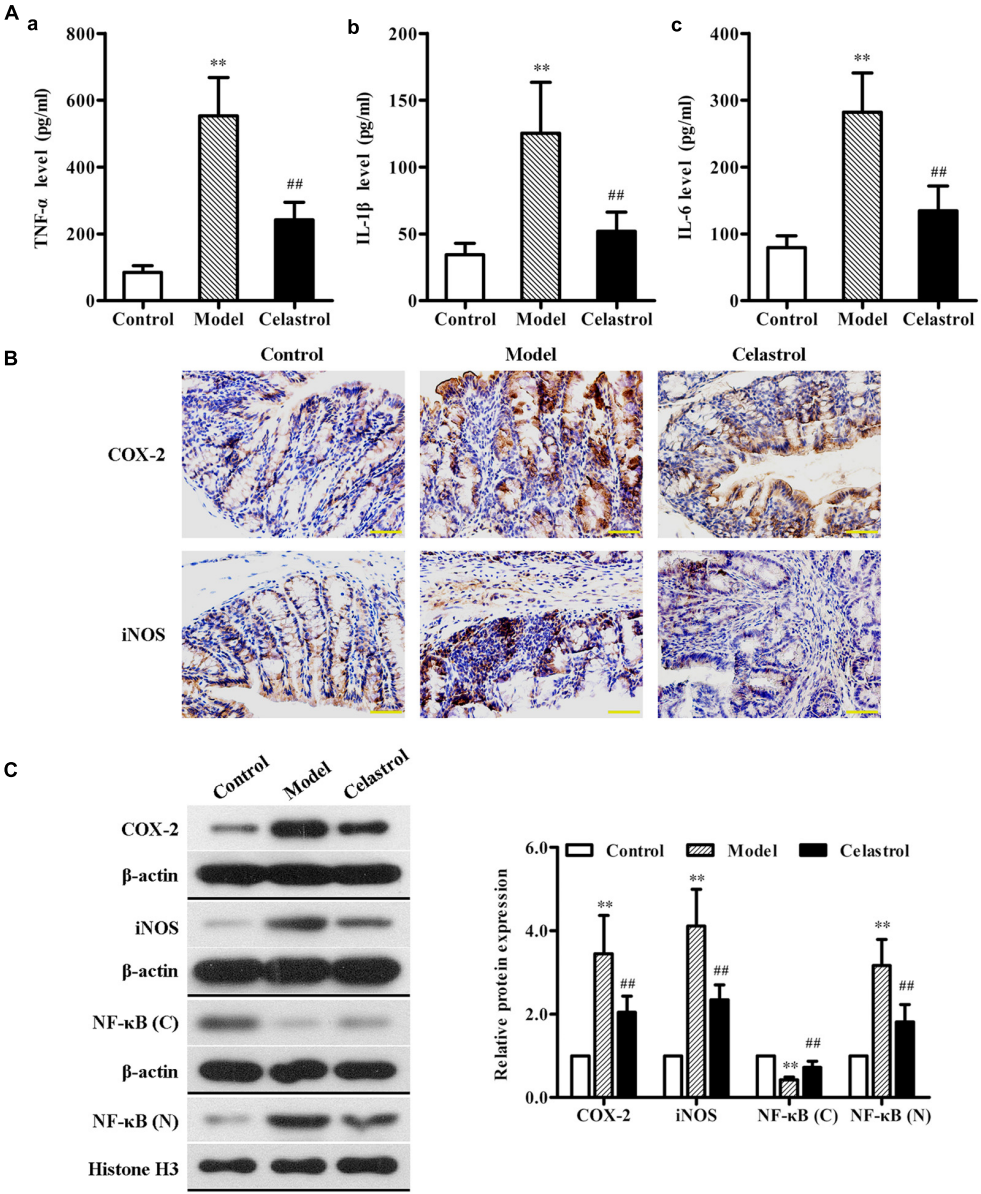
**Effects of celastrol (2 mg/kg) on the levels of inflammation cytokines and proteins in AOM/DSS-treated mice.** **(A)** The levels of tumor necrosis factor-α (TNF-α) (a), interleukin (IL)-1β (b), and IL-6 (c) in serum from the control mice, AOM/DSS model mice and AOM/DSS in combination with celastrol treated mice were determined using ELISA assay. **(B)** The expression levels of cyclooxygenase-2 (COX-2) and inducible nitric oxide synthase (iNOS) in normal colonic tissues or colonic tumor tissues was evaluated using immunohistochemical staining. Representative stained colonic sections from each group are shown. Original magnification was 400×. **(C)** The expression levels of COX-2 and iNOS as well as cytoplasmic and nuclear NF-κB p65 in colonic tissues from each group were determined by western blot analysis. Representative bands are shown (left), and the relative band intensity ratio was analyzed (right). *C*, cytoplasm; *N*, nuclear. Data are presented as mean ± SD (*n* = 6). ^∗∗^*p* < 0.01 vs. the control group; ^##^*p* < 0.01 vs. the AOM/DSS model group.

Overproduction of pro-inflammatory cytokines such as TNF-α, IL-1β, and IL-6 by activated macrophages plays an important role in the pathogenesis of UC (Murano et al., 2000). As illustrated in **Figure 4A**, the levels of serum TNF-α, IL-1β, and IL-6 in the AOM/DSS model group were significantly higher than in control group, as assessed by ELISA. Such increase in the levels of these inflammatory makers induced by AOM/DSS was attenuated by treatment with celastrol.

COX-2 and iNOS are two pro-inflammatory enzymes which are considered to be vital in the pathological process of UC (Dudhgaonkar et al., 2007). Additionally, iNOS acts in synergy with COX-2 to promote the inflammatory response (Sklyarov et al., 2011). Therefore, we evaluated the effects of celastrol on COX-2 and iNOS protein expression in the colonic tissue of AOM/DSS-induced mice. The results of immunochemical and western blot analyses showed that exposure of mice to AOM/DSS led to a significant increase in the expression of COX-2 and iNOS compared with the untreated mice. Oral administration of celastrol was able to obviously reduce the up-regulation of both pro-inflammatory proteins (**Figures 4B,C**).

Nuclear factor κB, a key transcription factor that mediates inflammatory signaling pathways, also plays a critical role in the pathophysiology of UC and CRC (Atreya et al., 2008; Paradisi et al., 2009). Normally, NF-κB is localized to the cytoplasm in an inactive form. During inflammatory stimulus, NF-κB is activated and translocates into the nucleus where it regulates the transcription of multiple genes involved in inflammatory response (Karrasch and Jobin, 2008). In order to evaluate whether celastrol also has an effect on NF-κB activation in our animal model of UC-CRC, the cytoplasmic levels and the nuclear levels of NF-κB p65 protein in colon tissues were determined, respectively, using western blot analysis. As shown in **Figure 4C**, compared with control group, the expression of NF-κB p65 protein in the cytoplasm was significantly decreased, whereas the nuclear NF-κB p65 levels were obviously increased in the AOM/DSS-treated model group, suggesting that NF-κB pathway may undergo activation. Nevertheless, celastrol reversed the decrease of cytoplasmic p65 protein and the increase of nuclear p65 protein induced by AOM/DSS. These results indicate that celastrol may suppress AOM/DSS-mediated activation of NF-κB signaling.

### Celastrol Inhibits AOM/DSS-Induced EMT

**FIGURE 5:**
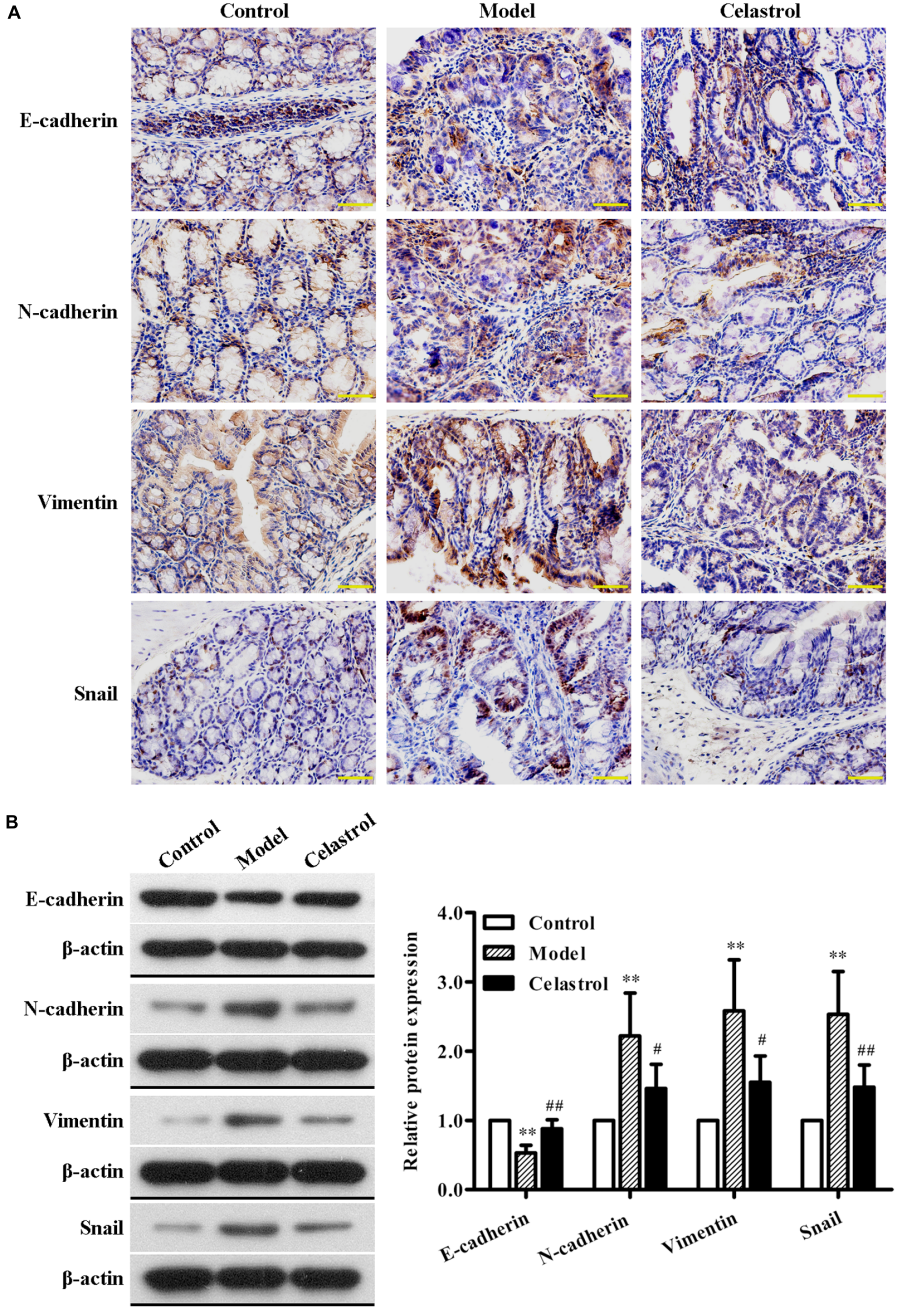
**Effects of celastrol (2 mg/kg) on the expression levels of EMT-related proteins in AOM/DSS-treated mice.** **(A)** The expression levels of E-cadherin, N-cadherin, Vimentin, and Snail in normal colonic tissues or colonic tumor tissues was assessed using immunohistochemical staining. Representative colonic sections from the control group, UC-CRC model group and celastrol-treated group are shown. Original magnification was 400×. **(B)** The expression levels of E-cadherin, N-cadherin, Vimentin, and Snail in colonic tissues from each group were determined by western blot analysis. Representative bands are shown (left), and the relative band intensity ratio was analyzed (right). Data are presented as mean ± SD (*n* = 6). ^∗∗^*p* < 0.01 vs. the control group; ^#^*p* < 0.05 and ^##^*p* < 0.01 vs. the AOM/DSS model group.

A hallmark of EMT is down-regulation of epithelial marker E-cadherin and up-regulation of mesenchymal markers N-cadherin and Vimentin, which is characterized by the loss of cell–cell adhesion and the gain of migratory and invasive phenotype (Yang et al., 2014). Snail, a zinc finger transcription factor, has been proved as a key regulator for EMT induction in CRCs (Nieto, 2002; Vandewalle et al., 2009). It is demonstrated that Snail suppresses E-cadherin transcription by binding to the E-box site within its promoter, resulting in EMT (Peinado et al., 2007). In this study, the expression of EMT regulatory proteins in the colon tissue was detected with immunohistochemical staining and western blot analysis. As shown in **Figure 5A**, significant down-regulation of E-cadherin and up-regulation of N-cadherin, Vimentin, and Snail were observed in AOM/DSS-induced UC-CRC mice compared with control group, suggesting the occurrence of EMT in the model group. Celastrol treatment was shown to dramatically increase the expression of E-cadherin and decrease the expression of N-cadherin, Vimentin, and Snail. Similarly, western blot analysis further confirmed the effects of celastrol on the expression levels of E-cadherin, N-cadherin, Vimentin, and Snail (**Figure 5B**). Taken together, these observations suggest that celastrol can repress EMT in UC-CRC model.

### Celastrol Inhibits Proliferation and EMT of HCT116 and HT-29 Cells

**FIGURE 6:**
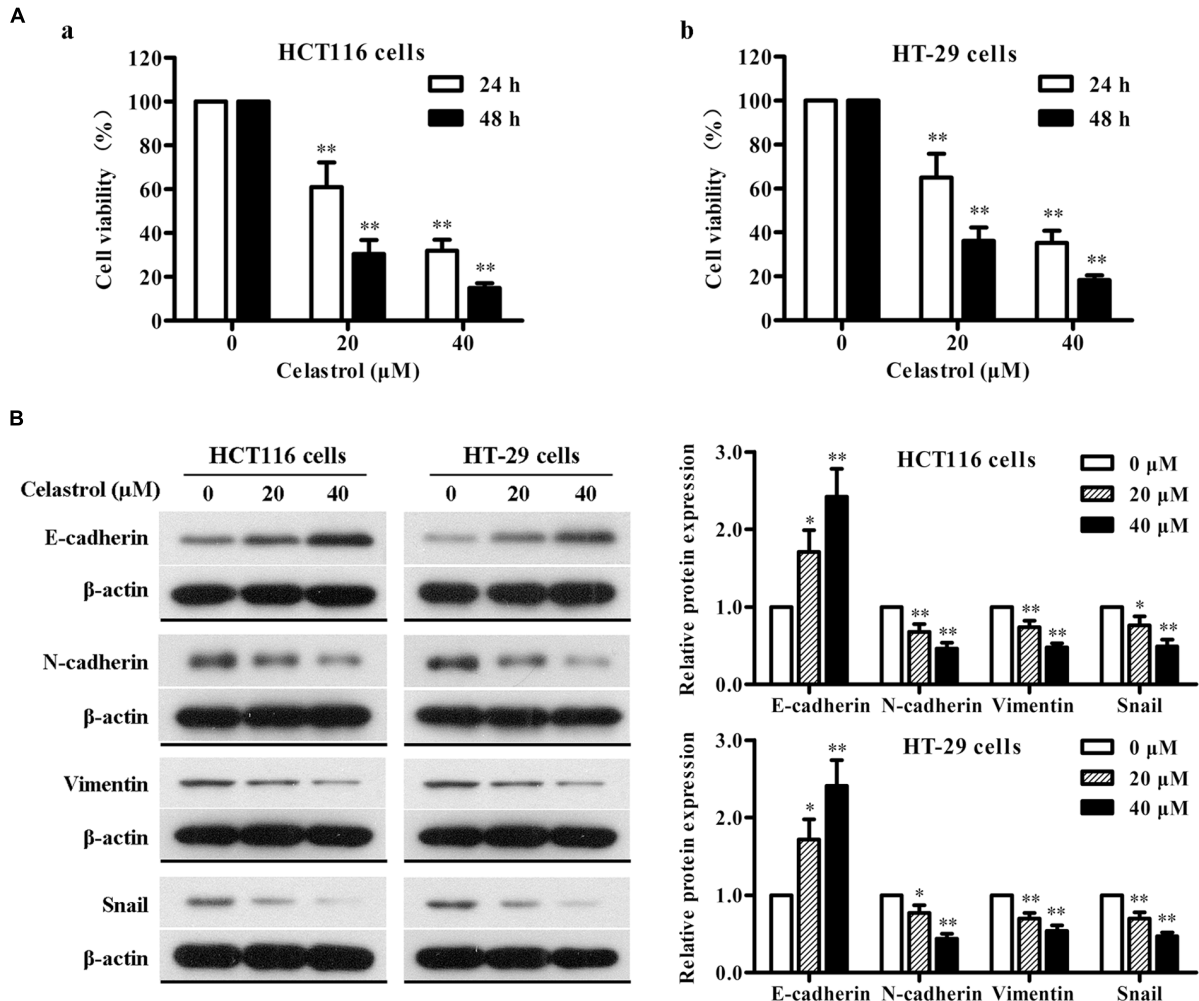
**Effects of celastrol on cell proliferation and EMT-related protein expression in CRC cells *in vitro*.** **(A)** HCT116 (a) and HT-29 (b) cells were exposed to celastrol (0, 20, and 40 μM) for 24 and 48 h, and cell viability was determined by MTT assay. **(B)** The expression levels of E-cadherin, N-cadherin, Vimentin, and Snail in HCT116 and HT-29 cells treated with celastrol (0–40 μM) for 48 h were determined by western blot analysis. Representative bands are shown (left), and the relative band intensity ratio was analyzed (right). Data are presented as mean ± SD from three independent experiments. ^∗^*p* < 0.05 and ^∗∗^*p* < 0.01 vs. control (0 μM).

The effect of celastrol on the viability of CRC cells HCT116 and HT-29 was determined using MTT assay. As shown in **Figure 6A**, celastrol treatment led to a significant reduction of cell viability in concentration- and time-dependent manner. It is demonstrated that the inhibitory rates were more than 80% after the treatment with 40 μM celastrol for 48 h in both cell lines. These findings indicate that celastrol is a potent inhibitor of CRC cell proliferation.

To gain further insight into the effect of celastrol on EMT in CRC, we determined the changes in expression of epithelial and mesenchymal markers in CRC cells with celastrol treatment for 48 h by western blot analysis. As shown in **Figure 6B**, celastrol significantly increased the expression of epithelial characteristic E-cadherin and decreased the expression of mesenchymal characteristics N-cadherin and Vimentin in both HCT116 and HT-29 cell lines. The transcription factor Snail was also down-regulated in a concentration-dependent manner. Therefore, these results suggest that celastrol can ameliorate EMT of CRC cells.

### Celastrol Inhibits Tumor Growth and EMT in Murine Models of Xenograft Tumor

**FIGURE 7:**
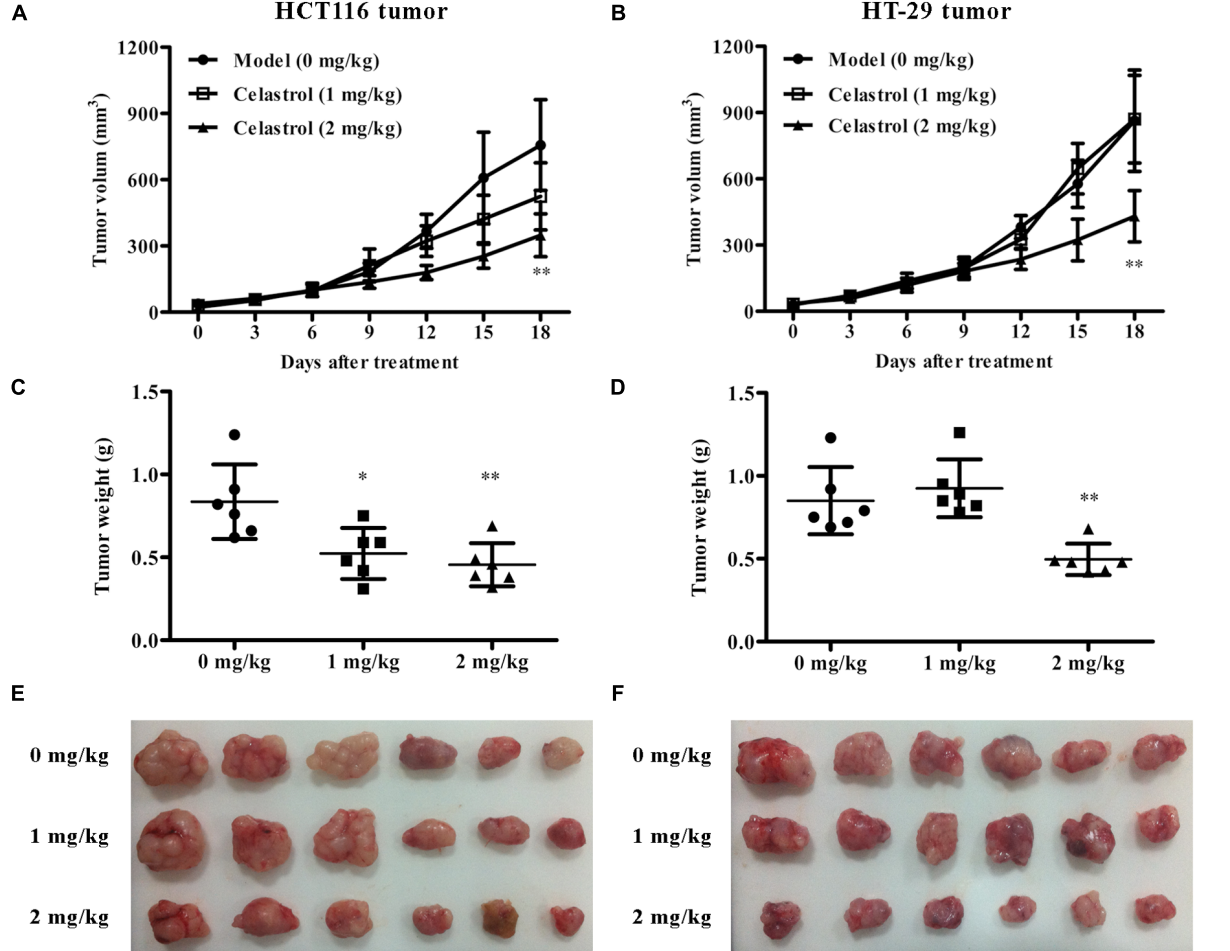
**Effects of celastrol on tumor growth in xenograft CRC models *in vivo*.** **(A,B)** Effect of celastrol (1 and 2 mg/kg) on tumor volumes in nude mice bearing HCT116 tumors **(A)** and HT-29 tumors **(B)**. **(C,D)** Effect of celastrol on tumor weight in each mouse bearing HCT116 tumors **(C)** and HT-29 tumors **(D)**. **(E,F)** The image of tumor tissues from each mouse bearing HCT116 tumors **(E)** and HT-29 tumors **(F)**. Data are presented as mean ± SD (*n* = 6). ^∗^*p* < 0.05 and ^∗∗^*p* < 0.01 vs. the model group (0 μM).

To test the *in vivo* anti-tumor efficacy of celastrol, we established nude mice models bearing inoculated HCT116 and HT-29 tumors. Remarkably, in both CRC models, mice treated with celastrol (2 mg/kg) displayed attenuated tumor growth compared with untreated mice (**Figures 7A,B**). The overall size and weight of the tumors in the celastrol-treated groups (2 mg/kg) was obviously lower than that of model group (**Figures 7C–F**). Analysis of tumor weights revealed that the inhibitory rates for HCT116 and HT-29 xenograft mice treated with 2 mg/kg celastrol were 45.5 and 41.6%, respectively. Throughout the treatment schedule, there was no significant difference in mean body weight between celastrol-treated mice and untreated mice (data not shown).

**FIGURE 8:**
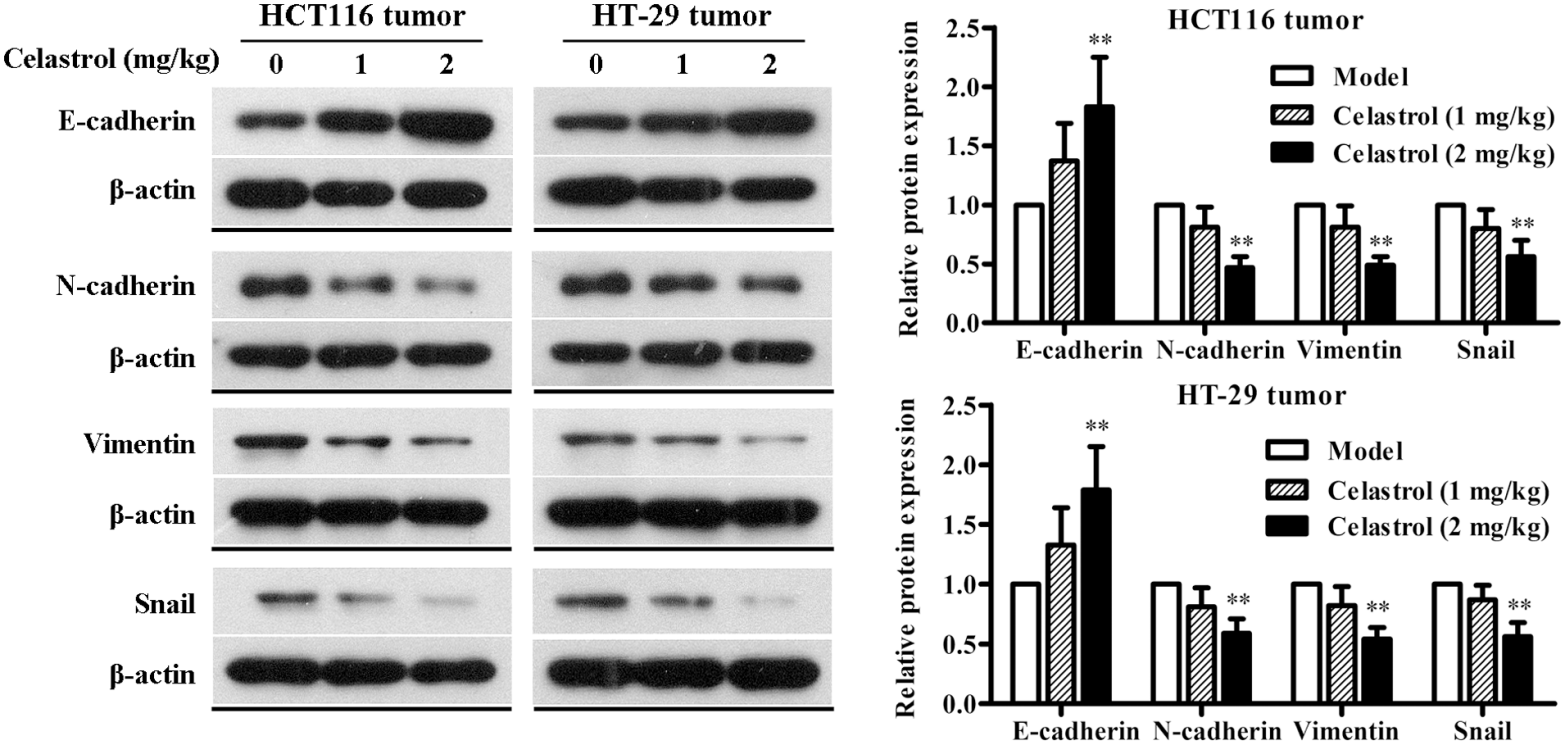
**Effects of celastrol on the expression levels of EMT-related proteins in xenograft CRC mice.** The expression levels of E-cadherin, N-cadherin, Vimentin, and Snail in HCT116 tumors and HT-29 tumors from each group were determined by western blot analysis. Representative bands are shown **(Left)**, and the relative band intensity ratio was analyzed **(Right)**. Data are presented as mean ± SD (*n* = 6). ^∗∗^*p* < 0.01 vs. the model group (0 μM).

We also investigated the effect of celastrol on EMT in nude mice with CRC and the results were consistent with that of CRC cells. As shown in **Figure 8**, the expression of E-cadherin was obviously up-regulated and the expression of N-cadherin, Vimentin, and Snail was significantly down-regulated in mice administered with 2 mg/kg celastrol compared to others. Collectively, these data further confirm that celastrol exhibits potent anti-tumor efficacy on CRC by down-regulating EMT.

## Discussion

Ulcerative colitis-related CRC is an irreversible malignant colonic disease with high mortality for which there is no effective therapies capable of curing or at least preventing the progressive course. Currently, increasing interest is being focused on exploring underlying mechanisms involved in UC-CRC and novel potential agents in animal models. During the preclinical study, AOM/DSS-induced model is the most commonly used non-hereditary UC-CRC mouse model, which can mimic the development of CRC in human patients. Based on the previous reports (Okayasu et al., 1996; Greten et al., 2004), we established a UC-CRC mouse model by three cycles of DSS administration in combination with AOM pretreatment, which led to 100% incidence of colonic neoplasms as well as marked symptoms including body weight loss, colon weight increase, and colon length shortening. In this study, we found that celastrol treatment significantly reduced the number of colonic neoplasms and tumor area, improved the above symptoms, and increased the survival rate of AOM/DSS-treated mice. In addition, celastrol was also shown to inhibit CRC cell proliferation and attenuate tumor growth in xenograft CRC models. These results suggest that celastrol can be considered as a potential therapeutic drug in ameliorating UC-CRC.

UC-CRC is a well-known multistep process during which the epithelial cells in colon undergo inflammation-dysplasia-carcinoma. An inflammatory environment is considered to play a key role in the initial stage of pathogenesis of UC-CRC. A large body of evidence suggests that activation of NF-κB is strongly induced in the inflamed colon from UC-CRC patients as well as in experimental UC-CRC models (Wullaert et al., 2011; Lin et al., 2014). The nuclear translocation of NF-κB can lead to increased levels of certain pro-inflammatory cytokines, such as TNF-α, IL-1β, and IL-6 in patients with UC (Ogata and Hibi, 2003). NF-κB activation can also promote expression of pro-inflammatory mediators including COX-2 and iNOS, which further deteriorates inflammatory responses and subsequently results in damage to the colonic tissues (Sakthivel and Guruvayoorappan, 2013). As to the effects of celastrol on inflammatory mediators in serum and colon tissues, we found that celastrol obviously decreased the overproduction of serum TNF-α, IL-1β, and IL-6, down-regulated the overexpression of COX-2 and iNOS proteins, and inhibited the activation and nuclear translocation of NF-κB in UC-CRC mice. These findings were in agreement with the previous studies supporting the notion that celastrol functioned as a potent NF-κB inhibitor in different *in vivo* and *in vitro* models for inflammation and cancer diseases (Pinna et al., 2004; Kannaiyan et al., 2011; Shaker et al., 2014).

There is increasing evidence supporting the promotional role of EMT in UC-CRC progression, which is associated with the loss of adhesive constraints, enhanced motility, the acquisition of stem cell-like properties, and immune escape (Bates, 2005; Zhu et al., 2013). Among a group of regulators involved in EMT, Snail has been identified as a central mediator of EMT by directly down-regulating E-cadherin in the progression of CRC (Fan et al., 2012). These findings imply that inhibiting EMT may be an ideal strategy for the treatment of UC-CRC. In a recent study, Kang et al. showed that celastrol could markedly inhibit TGF-β1-mediated EMT through regulating the expression of Snail and E-cadherin in Madin-Darby Canine Kidney (MDCK) and A549 cell lines (Kang et al., 2013). However, the possible effect of celastrol on EMT in UC-CRC model is unclear. As expected, our data demonstrated that the down-regulated E-cadherin as well as the up-regulated N-cadherin, Vimentin, and Snail by AOM/DSS could be inhibited by celastrol treatment. Moreover, the suppression of EMT was also involved in the inhibitory roles of celastrol in human CRC cell proliferation *in vitro* (HCT116 and HT-29 cells) and xenograft colonic tumor growth *in vivo*. Therefore, the results presented here suggest that the alleviative effect of celastrol on AOM/DSS-induced UC-CRC may be partially mediated by suppressing EMT, although the detailed mechanisms need to be explored.

P53 is a tumor suppressor protein which plays an important role in cell cycle, DNA repair, apoptosis, senescence, and angiogenesis (Sionov and Haupt, 1999). Increasing evidence indicates that p53 mutations and loss of heterozygosity are the early events during the progression of UC-CRC (Garrett et al., 2009; Scarpa et al., 2014). It is interesting that wild-type p53 protein has a short half-life and cannot be determined using immunohistochemical staining, therefore, the positive p53 immunochemical results denote mutated p53 protein (Seyedmajidi et al., 2013). Here, we found that celastrol treatment remarkably down-regulated the expression of the dysfunctional p53 and p-p53. Moreover, β-catenin and PCNA, as two important oncogenic transcription factors, have also been reported to play crucial roles during UC-associated colon carcinogenesis (Cappello et al., 2003; Nelson and Nusse, 2004; Clevers, 2006; Georgescu et al., 2007). Our results clearly demonstrated that the expression levels of β-catenin and PCNA in colonic tissues were significantly up-regulated by AOM/DSS, which was in line with previous studies (Lin et al., 2014, 2015). Nevertheless, administration of celastrol significantly prevented up-regulation of these neoplastic markers. These data reveals that celastrol could execute its protective effect against UC-CRC by preventing the process of carcinogenesis.

During the progression of UC-CRC, there is abundant evidence for the complex relationship among inflammation, EMT, and carcinogenesis. Several inflammatory mediators, such as TNF-α, IL-6, TGF-β, and NF-κB have been reported to be involved in the whole progression including triggering inflammatory cascade, promoting EMT and facilitating cell transformation and malignancy (Landskron et al., 2014). β-catenin, an important downstream regulator of the Wnt signaling pathway, also participates in EMT procession by binding to membranous E-cadherin (Munding et al., 2012). Our present findings suggest that celastrol can prevent the development of UC-CRC in mice via targeting multiple mechanisms across the pathological progression, including alleviating inflammation, intervening EMT, and suppressing carcinogenesis.

## Conclusion

Our studies demonstrated for the first time that celastrol could effectively prevent UC-related colonic carcinogenesis in AOM/DSS mice model. The mechanisms involved in this effect of celastrol on UC-CRC were associated with suppression of inflammatory responses, intervention of EMT as well as down-regulation of mutated p53 and p-p53 proteins, oncogenic proteins β-catenin, and PCNA. Furthermore, the effect of celastrol on EMT reversal was also confirmed in CRC cells *in vitro* and the colon cancer xenograft *in vivo*. Based on the data presented here, we believe that celastrol may be a potential therapeutic agent for UC-CRC treatment and the research on its more precise mechanisms is ongoing in our group.

## Author Contributions

Conceived and designed the experiments: LL, YS, and SZ. Performed the experiments: LL, YS, DW, SZ, JZ, and CZ. Analyzed and interpreted the data: LL, YS, DW, SZ, and JZ. Drafted the paper and revised it critically for important intellectual content: LL, YS, and DW.

## Conflict of Interest Statement

The authors declare that the research was conducted in the absence of any commercial or financial relationships that could be construed as a potential conflict of interest.

The reviewer (EP) and handling Editor (AI) declared their shared affiliation, and the handling Editor states that the process nevertheless met the standards of a fair and objective review.
